# Supplementary material for: Biocontrol Potential of the New Codling Moth Granulovirus (CpGV) Strains
Source: Microorganisms. 2024 Sep 30;12(10):1991. doi: 10.3390/microorganisms12101991 (PMC11510065; doi:10.3390/microorganisms12101991)
Supplement: Supplementary file 1 [file microorganisms-12-01991-s001.zip › Supplementary Table S1 Viruses.docx]

**Supplementary Table S1.** Place of isolation and year of the CpGV strains introduction into the BRC of the FSBSI FRCBPP

| **№** | **Strain/code** | **Place of isolation** | **Year of introduction**  **into the collection** |
| --- | --- | --- | --- |
| 1 | BZR GV 1 | - | 2018 |
| 2 | BZR GV 2 | Farm Olzhans, Kastanay region, rep. Kazakhstan | 2018 |
| 3 | BZR GV 3 | Farm Olzhans, Kastanay region, rep. Kazakhstan | 2018 |
| 4 | BZR GV 4 | FSBSI FRCBPP, Krasnodar, Russia | 2018 |
| 5 | BZR GV 5 | FSBSI FRCBPP, Krasnodar, Russia | 2018 |
| 6 | BZR GV 6 | FSBSI FRCBPP, Krasnodar, Russia | 2018 |
| 7 | BZR GV 7 | FSBSI FRCBPP, Krasnodar, Russia | 2018 |
| 8 | BZR GV 8 | FSBSI FRCBPP, Krasnodar, Russia | 2018 |
| 9 | BZR GV 9 | FSBSI FRCBPP, Krasnodar, Russia | 2018 |
| 10 | BZR GV 10 | LLC "Red Garden", Rostov region, Russia | 2018 |
| 11 | BZR GV 12 | FSBSI FRCBPP, Krasnodar, Russia | 2019 |
| 12 | BZR GV 13 | FSBSI FRCBPP, Krasnodar, Russia | 2019 |
| 13 | BZR GV L-2 | - | 2018 |
| 14 | BZR GV L-4 | - | 2018 |
| 15 | BZR GV L-5 | - | 2018 |
| 16 | BZR GV L-6 | - | 2018 |
| 17 | BZR GV L-7 | - | 2018 |
| 18 | BZR GV L-8 | - | 2018 |
