# Supplementary material for: Biocontrol Potential of the New Codling Moth Granulovirus (CpGV) Strains
Source: Microorganisms. 2024 Sep 30;12(10):1991. doi: 10.3390/microorganisms12101991 (PMC11510065; doi:10.3390/microorganisms12101991)
Supplement: Supplementary file 1 [file microorganisms-12-01991-s001.zip › Supplementary Table S2 Viruses.docx]

**Supplementary Table S2.** Inventory numbers of CpGV strains sequences from the BRC of the FSBSI FRCBPP deposited in NCBI.

| **№** | **Strain/code** | **Id of the sequence in the genome assembly** | **Sequence inventory number in NCBI** |
| --- | --- | --- | --- |
| 1 | BZR GV 1 | id=1  id=2  id=3  id=4 | OR743626  OR743627  OR743628  OR743629 |
| 2 | BZR GV 2 | id=1  id=2  id=3  id=4  id=5  id=6  id=7  id=8  id=9  id=10  id=11  id=12  id=13  id=14  id=15  id=16  id=17  id=18  id=19  id=20  id=21  id=22  id=23  id=24  id=25  id=26  id=27  id=28 | OR743630  OR743631  OR743632  OR743633  OR743634  OR743635  OR743636  OR743637  OR743638  OR743639  OR743640  OR743641  OR743642  OR743643  OR743644  OR743645  OR743646  OR743647  OR743648  OR743649  OR743650  OR743651  OR743652  OR743653  OR743654  OR743655  OR743656  OR743657 |
| 3 | BZR GV 3 | id=1 | OR743658 |
| 4 | BZR GV 4 | id=1_gv4  id=2_gv4 | OR743675  OR743676 |
| 5 | BZR GV 5 | id=1_gv5  id=2_gv5  id=3_gv5  id=4_gv5 | OR743677  OR743678  OR743679  OR743680 |
| 6 | BZR GV 6 | id=1  id=2  id=3  id=4  id=5  id=6 | OR743681  OR743682  OR743683  OR743684  OR743685  OR743686 |
| 7 | BZR GV 7 | id=1_gv7  id=2_gv7 | OR743687  OR743688 |
| 8 | BZR GV 8 | id=1_gv8  id=2_gv8 | OR743689  OR743690 |
| 9 | BZR GV 9 | id=1_gv9  id=2_gv9 | OR743691  OR743692 |
| 10 | BZR GV 10 | id=1_gv10  id=2_gv10  id=3_gv10 | OR743693  OR743694  OR743695 |
| 11 | BZR GV 12 | id=1  id=2  id=3  id=4  id=5  id=6 | OR743696  OR743697  OR743698  OR743699  OR743700  OR743701 |
| 12 | BZR GV 13 | id=1_gv13  id=2_gv13 | OR743702  OR743703 |
| 13 | BZR GV L-2 | Seq1_L2  Seq2_L2 | OR743659  OR743660 |
| 14 | BZR GV L-4 | Seq1_L4  Seq2_L4  Seq3_L4  Seq4_L4 | OR743661  OR743662  OR743663  OR743664 |
| 15 | BZR GV L-5 | Seq1_L5  Seq2_L5  Seq3_L5 | OR743665  OR743666  OR743667 |
| 16 | BZR GV L-6 | Seq1_L6 | OR743668 |
| 17 | BZR GV L-7 | Seq1_L7  Seq2_L7 | OR743669  OR743670 |
| 18 | BZR GV L-8 | Seq1_L8  Seq2_L8  Seq3_L8  Seq4_L8 | OR743671  OR743672  OR743673  OR743674 |
